# Disparities between malaria infection and treatment rates: Evidence from a cross-sectional analysis of households in Uganda

**DOI:** 10.1371/journal.pone.0171835

**Published:** 2017-02-27

**Authors:** Indrani Saran, Jessica Cohen

**Affiliations:** 1 Duke Global Health Institute, Duke University, Durham, NC, United States of America; 2 Department of Global Health and Population, Harvard T.H. Chan School of Public Health, Boston, MA, United States of America; Centro de Pesquisas Rene Rachou, BRAZIL

## Abstract

**Background:**

In Sub-Saharan Africa, both under-treatment and over-treatment of malaria are common since illnesses are often diagnosed and treated on the basis of symptoms. We investigate whether malaria treatment rates among febrile individuals correspond to observed patterns of malaria infection by age and by local prevalence.

**Methods and findings:**

We use data on treatment of febrile illnesses from a household survey that was conducted between March and May 2012 in 92 villages in six districts in Eastern Uganda. All household members were also tested for malaria using a rapid diagnostic test. We show that both the age of the febrile individual and the village prevalence rate are strongly associated with the odds that a febrile patient was infected with malaria, but not with the odds of ACT treatment. Compared to individuals who were aged 15 or above, febrile individuals aged 5–14 had 3.21 times the odds of testing positive for malaria (95% CI: [2.36 4.37], P<0·001), and febrile individuals who were under age 5 had 2.66 times the odds of testing positive for malaria (95% CI: [1.99 3.56], P<0·001). However, ACT treatment rates for febrile illnesses were not significantly higher for either children ages 5–14 (Unadjusted OR: 1.19, 95% CI: [0.88 1.62], P = 0.255) or children under the age of 5 (Unadjusted OR: 1.24, 95% CI: [0.92 1.68], P = 0·154). A one standard deviation increase in the village malaria prevalence rate was associated with a 2.03 times higher odds that a febrile individual under the age of five tested positive for malaria (95% CI: [1.63 2.54], p<0·001), but was not significantly associated with the odds of ACT treatment (Un-adjusted OR: 0.83, 95% CI: [0.66 1.05], P = 0·113). We present some evidence that this discrepancy may be because caregivers do not suspect a higher likelihood of malaria infection, conditional on fever, in young children or in high-prevalence villages.

**Conclusion:**

Our findings suggest that households have significant mis-perceptions about malaria likelihood that may contribute to the under-treatment of malaria. Policies are needed to encourage caregivers to seek immediate diagnostic testing and treatment for febrile illnesses, particularly among young children.

## Introduction

Malaria continues to pose a large morbidity and mortality burden with an estimated 214 million cases in 2015 and nearly 440,000 deaths [1]. A majority of these deaths occur in Sub-Saharan Africa, primarily among young children, and could likely be avoided with prompt treatment of the illness with artemisinin-based combination therapies (ACTs), the first-line treatment for uncomplicated malaria [1,2].

In most countries, ACTs are freely available at public sector health facilities, and there are efforts to make them available at subsidized prices in the private sector, where many people first seek care for suspected malaria [3]. However, it is estimated that only 12–22% of malaria cases in children under age five were treated with ACTs in 2014. This low level of coverage with appropriate treatment is partly due to the fact that nearly 45% of children with fever (the primary symptom of malaria) either did not get any care outside the home or sought care in the informal private sector, where they are less likely to get ACTs than if they sought public sector treatment [1]. Even when patients do use public health facilities, they do not always receive ACTs [1,4].

At the same time, many individuals who do *not* have malaria are treated with ACTs. Malaria is often diagnosed and treated on the basis of symptoms such as fever, chills, headache, fatigue, and nausea, which overlap with several other common bacterial and viral illnesses such as pneumonia or the flu [5,6]. The World Health Organization (WHO) now recommends universal diagnostic testing for malaria, and diagnostic confirmation of malaria cases in the African public health sector has increased from 36% to over 60% between 2005 and 2014 [1]. However ACT treatment for non-malarial illnesses is still common, partly because many individuals seek care in the private sector where diagnostic testing is very limited, but also because health workers, caregivers and sick individuals often treat illnesses with ACTs even if the individual tests negative for malaria [7–9].

Although it is difficult to identify malaria cases on the basis of symptoms alone, the age of the individual and the local malaria prevalence are strong predictors of malaria infection status [10,11]. For example, a review of studies across sub-Saharan Africa finds that the median proportion of fevers attributable to malaria was 36% among children under five years and 26% for those aged five and above[12]. Another study estimates that, across Africa, only 3.3% of febrile children presenting at clinics in low malaria prevalence areas were infected with malaria, but 59% of febrile children in high prevalence areas had malaria [13]. Models of malaria transmission predict that, for children under the age of five, clinical incidence of malaria is strongly increasing with prevalence [14,15]

In this study we investigate whether ACT treatment patterns for febrile illnesses correspond to patterns of malaria infection by patient age and by local prevalence. We focus on age and prevalence as these are two factors that have been shown to be strongly correlated with the probability of malaria infection in febrile patients. We hypothesized that patterns of ACT treatment are related to household beliefs about the likelihood of a febrile illness being malaria. In order to test this, we also examine how malaria beliefs vary with the age of the febrile individual and with local prevalence. In addition, we directly examine the association between malaria beliefs and ACT treatment and compare this to the association between other household, respondent, and febrile individual characteristics hypothesized to be important for ACT treatment. We use data from a unique household survey in Eastern Uganda that asked about household malaria treatment behavior and malaria beliefs, and also included a malaria rapid diagnostic test of all household members. This study provides evidence on the extent to which household beliefs about malaria may contribute to the under-treatment of malaria.

## Methods

### Ethics statement

Ethical approval for this study was granted by the Harvard School of Public Health Internal Review Board (Protocol# P19371-105) and the Uganda National Council for Science and Technology (Protocol # HS805). Written consent was obtained from all interviewed respondents.

### Study context and population

This study used data from a household survey that was conducted over 9 weeks between March and May 2012 in 92 villages in six districts in Eastern Uganda: Budaka, Bukedea, Kibuku, Kumi, Ngora and Pallisa. Malaria is highly endemic in this region with over 100 infective bites per person per year and peaks in transmission during the rainy seasons which occur in the months of March through May and September to December [16].

The household survey was conducted as part of a randomized controlled trial that tested the feasibility and impact of introducing rapid diagnostic tests for malaria in private sector drug shops, described in more detail in previously published work [9,17]. 2,285 households were visited for a baseline survey in March and April 2011 and were visited again for monthly follow-up surveys for nine months to ask about illnesses household members experienced in the month prior to the survey. This analysis used only the last survey round, when all consenting adults and all children whose parents or caregivers gave consent for them, were tested for malaria using a rapid diagnostic test (RDT). The RDT used in this study was the CareStart Malaria HRP2 (Pf) test (Access Bio, Somerset, USA). This test has a panel detection score of 98.7%, a false-negative rate of < 1% and a total false-positive rate of 2.4% [18].

### Data

The survey asked the female household head (i.e. the female primarily responsible for household health decisions) to list illnesses that any member of the household had in the month prior to the survey, including the start and end date. For children under the age of 18, in 75% of cases the respondent was the mother of the patient and in 13% of cases she was the grandparent (in the remaining cases, the respondent was another relative, like an aunt). The respondent was questioned about the symptoms experienced, and was also specifically asked if the sick individual had any of a number of symptoms including fever, chills, headache, joint pain, loss of appetite, diarrhea, vomiting, stomach pains and runny nose/congestion. The respondent was then asked about the sequence of treatments sought for the illness, what drugs were obtained, whether the sick individual was tested for malaria, and the result of the test. Respondent perceptions of the likelihood that the illness was malaria were elicited using a visual analog ladder scale.

We supplemented this data on illness treatment with household demographic information, and with data from a census of all local public health facilities and licensed drug shops, also conducted as part of the main study, which included the GPS co-ordinates of the facilities and information about ACT stocks.

### Analytical approach

We investigated whether ACT treatment for febrile illnesses, and beliefs about whether a febrile illness was malaria, corresponded to patterns of malaria infection (as measured by the RDT) by the age of the febrile individual and by the village prevalence rate. We focused on febrile illnesses, as fever is the symptom most strongly associated with malaria and is also used as a WHO indicator of suspected malaria in malaria-endemic areas [1,2]. Since no formal definition of fever was given to the respondent, our analysis relied on febrile illnesses as self-reported by the respondent. Importantly, since the RDT was performed at the end of the survey, respondents did not know the RDT result of household members when responding to questions about ACT use and malaria likelihood.

The RDT identifies a malaria infection by detecting the presence of an antigen known as histidine-rich protein 2 (HRP) which can persist several weeks after the malaria infection has cleared [19]. Studies have found that 88–98% of individuals still have a positive RDT test result 14 days after beginning treatment [20–22]. The RDT, therefore, is not only a measure of a current infection, but can also detect one that may have been treated and cured up to two weeks before the test. Our sample, therefore, consisted of all febrile episodes that ended no more than 14 days prior to the date of the survey (which was also the date of the RDT). This methodology for measuring malaria infection and treatment rates is also used by the World Health Organization in the two most recent “World Malaria Reports” [1,23].

Our first outcome of interest was whether a febrile individual had clinical malaria. We defined a febrile illness as malaria if it ended no more than two weeks before the survey and the patient tested positive for malaria on the survey RDT.

Our second outcome of interest was whether the febrile individual was reported to have been treated with an ACT at any time during the illness episode. For each illness, the respondent was asked what drugs were used at home, and what drugs were given or purchased at a health facility or drug shop. A drug was coded as an ACT either if the respondent said it was an ACT, or gave the name or brand of a particular type of ACT (such as “Coartem” or “Lumartem”). We excluded from the analysis the 7 individuals who reported previously testing negative for malaria, but tested positive for malaria when we performed the RDT at the end of the survey. These individuals may have become infected between the time they sought treatment for their illness and the survey and their fever may have been due to another cause.

Our final outcome of interest was whether the respondent believed that the febrile individual’s illness was malaria. One measure of the respondent’s beliefs about whether the illness was malaria was if she mentioned “malaria” when asked what the febrile individual’s health problem was. However, some respondents simply gave symptoms in response to this question. For all febrile illnesses, respondents were also asked what they thought was the likelihood that the illness was malaria on a visual analog ladder scale from 0 (no chance of malaria) to 10 (definitely malaria). Therefore, we defined a respondent as believing an illness was malaria if she either reported the health problem as “malaria”, or if she gave a rating between 8 and 10 on the 0–10 scale. While the proportion of febrile illnesses perceived to be malaria was sensitive to the way this variable is defined, the patterns of beliefs by age of the individual, and by village prevalence, were generally similar regardless of how we defined a respondent’s beliefs about whether a febrile illness was malaria (S1 Fig). In all analyses that include respondents’ beliefs about whether the illness was malaria, we excluded the 165 febrile individuals who had already received a malaria test, as the respondents’ beliefs may have been influenced by the test result.

Our two independent variables were the age of the febrile individual and the village malaria prevalence rate. Prevalence was defined as the proportion of 2–10 year olds in the village who tested positive for malaria on the RDT (regardless of whether they reported any symptoms). We used the proportion of 2–10 year olds testing positive, instead of the proportion of all ages testing positive, as this is considered a stable measure of local prevalence [24]. We excluded the febrile individual’s own RDT status when calculating the village prevalence rate for each individual. Furthermore, when investigating the relationship between village prevalence and the outcomes of interest, we limited the sample to children under the age of five. For older ages, the relationship between malaria prevalence and clinical incidence is not straightforward because in high-prevalence areas, adults are likely to have higher natural immunity to the disease resulting from greater exposure to the parasite when young [15,25].

We provide graphical evidence to show how each of the outcomes vary with the age, and with the village malaria prevalence rate, of individuals who had a fever in the two weeks prior to the survey. We binned febrile individuals ages into 1-year age groups for individuals under the age of 5, and 5-year age groups for individuals aged 5 and above. For village prevalence rates—which ranged from 0.08 to 0.93—we divided them into 0.10 unit bins. The mean of the outcome (malaria positivity rates, ACT treatment rates, malaria beliefs) was calculated within each bin and plotted against febrile individual age and, separately, against village prevalence rates. On the same graphs, we also included a local polynomial or local linear regression line, and 95% confidence intervals, of the outcome on the independent variable of interest (for the regression line we do not bin age/village prevalence).

We used logistic regressions to test whether the graphical relationships were statistically significant and to control for other factors that might influence ACT use and also be associated with our variables of interest. The control variables, which have been shown to either influence treatment-seeking for febrile illnesses, or to be directly associated with the use of ACTs, included the education level of the respondent, whether he/she could read a basic letter in English, the wealth quintile of the household, the distance of the household to the closest health center, hospital, clinic, and drug shop, and whether ACTs were available at the closest licensed drug shop at the time of the survey [4,26–31]. We tested the equality of coefficients from separate models with either malaria positivity as an outcome or ACT treatment as an outcome using the “suest” command in STATA which estimates both models simultaneously.

In addition, we show both the bi-variate and multi-variate associations between ACT treatment—for both febrile illnesses and RDT-positive febrile illnesses—and respondents’ beliefs about whether the illness was malaria, the age of febrile individual, village prevalence and the control variables. For the multi-variate model, we also performed stepwise backward-selection estimation of a logistic model (using the “stepwise” command in STATA). The significance level for removal from the model was 0.2.

We defined a respondent as having some primary education if they completed part of, or all of, primary school (but had no further education). A respondent was defined as having some secondary education if they had any education beyond primary school. We assigned households to wealth quintiles using a principal component analysis of housing characteristics and household ownership of durable assets and farm animals [32]. Distance to the nearest health facility and drug shop was based on the GPS co-ordinates of the households and the health facilities, and was calculated using the length of the shortest curve between the two GPS co-ordinates along the surface of a mathematical model of the earth. A drug shop was defined as stocking ACTs if the drug shop attendant mentioned it as one of the five most frequently sold anti-malarial drugs. This variable was missing for 156 people in the sample. For 93 of these people, the missing value was imputed as the mean proportion of drug shops in the person’s village that had ACTs available. All regressions with ACT stocks also include an indicator variable to indicate which of these observations had missing values that were imputed.

In all regressions, standard errors were adjusted for clustering at the village level (using the STATA cluster command) as this was the level at which households were randomly sampled. All analyses were conducted using Stata/SE version 11 (StataCorp, College Station, TX) [33].

## Results

### Sample characteristics and malaria positivity rates

The survey was conducted with respondents from 2,285 households and included 9,887 individuals as members of those households. Table 1 presents some summary statistics on the 1,342 individuals who had a fever in the two weeks prior to the survey and on the respondents for these individuals. 20% of respondents had no education, while 62% had some primary education. Only 31% could read a simple letter in English. Febrile individuals were from households that were relatively poor: while 83% owned some land, only 5% had electricity, and 57% owned at least one mobile phone.

**Table 1:** Summary statistics of respondent, household, and febrile individual characteristics and treatment-seeking behavior.

	% or Mean ±SD	Observations
***A*. *Characteristics of Respondent***		
Age	35.0 ± 12.4	1288
Female	96%	1338
No Education	20%	1334
Some Primary Education	62%	1334
Some Secondary Education	17%	1334
Can Read English	31%	1342
***B*. *Characteristics of Household***		
Has Electricity	5%	1340
Owns Mobile Phone	57%	1342
Owns Land	83%	1342
Number of Household Members	7.33 ± 3.40	1342
Village Malaria Prevalence Rate in 2–10 year olds	63%	1342
Village Malaria Prevalence Rate among all ages	48%	1342
***C*. *Household Distance to Health Facilities***		
Distance to closest Health Center (km)	2.01 ± 1.81	1341
Distance to closest Hospital (km)	10.2 ± 5.67	1341
Distance to closest private clinic (km)	3.50 ± 3.34	1341
Distance to closest drug shop (km)	0.79 ± 0.91	1341
Distance to closest licensed drug shop (km)	1.61 ± 1.70	1341
ACTs available at closest licensed drug shop	82%	1279
***D*. *Characteristics of Febrile Individual***		
Age	13.9 ± 18.0	1312
Under 5	44%	1342
Female	58%	1342
Tested Positive for Malaria	52%	1342
***E*. *Treatment-Seeking for Febrile Illness***		
Ever Sought Care	58%	1342
Sought Care in Public Sector	20%	1342
Sought Care at Private Clinic	7%	1342
Sought Care at Drug Shop	33%	1342
***F*. *Malaria Testing and Treatment***		
Ever Tested at Health Facility/Drug Shop	12%	1335
Tested Positive for Malaria (Among Those Tested)	76%	172
Took ACT anywhere	42%	1342
Took ACT if Treated at Home	36%	559
Took ACT if Treated Outside Home	46%	783
Took ACT if Tested Positive at Health Facility/Drug Shop	54%	131
Took Other Anti-Malarial Drug	21%	1342
Respondent Reported Illness Was Malaria	34%	1342

Sample is limited to individuals who had a fever in the two weeks prior to the survey.

The mean village malaria prevalence rate was 63% in children aged 2–10 years. The distance to the closest health center was, on average, 2 km, while the mean distance to the closest drug shop (either licensed or unlicensed) was less than half of that at 0·8km. 82% of households had ACTs available at their closest licensed drug shop which was, on average, 1.6km away.

584 of the 1,342 (44%) febrile episodes were among children under the age of five. Among the 1,342 febrile episodes, 702 (52%) tested positive for malaria when they were tested at the end of the survey, and 343 children under the age of five (59%) tested positive for malaria at the end of the survey. 58% of febrile individuals received care from outside the home for their febrile illness, 33% went to a drug shop, while 20% were treated in the public sector.

Only 12% of febrile individuals had received a diagnostic test for malaria at either a health facility or drug shop. Of those tested, 76% reported a positive test, and 54% of those who said they tested positive reported taking an ACT. 36% of febrile individuals who did not seek care outside the home reported taking an ACT, while 46% of people who sought care outside the home said they took an ACT. Among all febrile individuals, 21% reported taking a non-ACT antimalarial. Respondents reported 34% of the febrile illnesses as malaria.

### Malaria positivity and ACT use by age of febrile individual and by village prevalence

**Fig 1:**
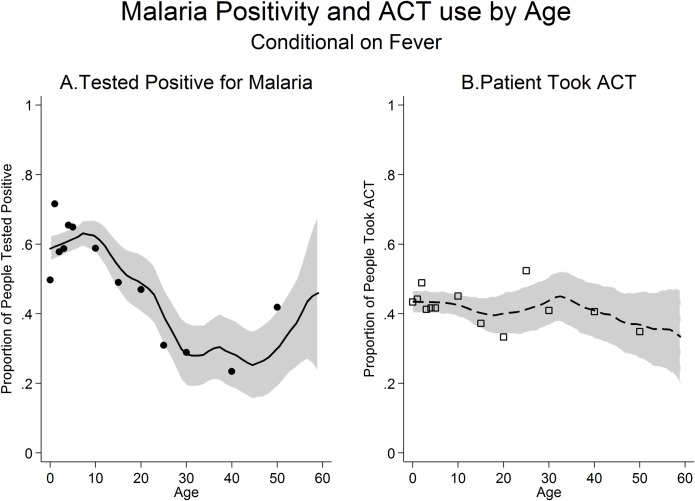
Malaria infection rates and ACT use by age. (A) The proportion of febrile individuals who tested positive for malaria on the RDT. (B) The proportion of febrile individuals who were treated with an ACT. Figure shows mean of the outcome within 1 year (for ages under 5) or 5-year (for ages 5 and above) age groups. A local polynomial regression line is also plotted and the grey shaded areas indicate 95% confidence intervals. Data is limited to individuals who had a fever in the two weeks prior to the survey. Ages above 60 are excluded because of small sample size.

Fig 1 shows how malaria infection rates and ACT use varied with the age of febrile individuals. The malaria positivity rate in febrile individuals declined substantially with age, from 60% to 30% between the ages of 0–45. Compared to febrile individuals who were aged 15 or above, febrile children aged 5–14 had 3.21 times the odds of testing positive for malaria (95% CI: [2.36 4.37], P<0·001), and febrile children who were under 5 had 2.66 times the odds of testing positive for malaria (95% CI: [1.99 3.56], P<0·001) with very similar effects when adjusted for covariates (S1 Table). However, febrile children were not comparatively more likely to be treated with an ACT than febrile individuals aged 15 or above. Compared to those aged 15 or above, the odds of being treated with an ACT were not significantly higher for children under the age of 5 (Unadjusted OR: 1.24, 95% CI: [0.92 1.68], P = 0·154; Adjusted OR: 1.20, 95% CI [0.87 1.64], P = 0·269) or children ages 5–14 (Un-adjusted OR: 1.19, 95% CI: [0.88 1.62], P = 0.255, Adjusted OR: 1.29, 95% CI: [0.94 1.77], P = 0.112) (S1 Table).

**Fig 2:**
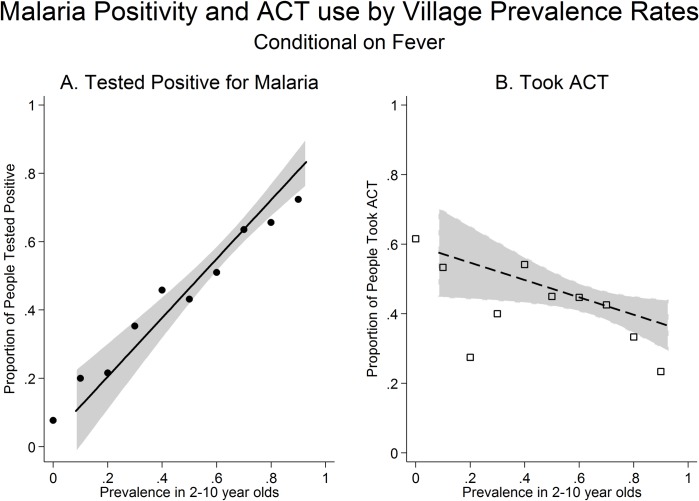
Malaria infection rates and ACT use by village prevalence. (A) The proportion of febrile individuals who tested positive for malaria on the RDT. (B) The proportion of febrile individuals who were treated with an ACT. Figure shows mean of the outcome within 0.10 units of village prevalence. A local linear regression line is also plotted and the grey shaded areas indicate 95% confidence intervals. Data is limited to individuals who had a fever in the two weeks prior to the survey and to individuals under the age of five.

Our sample consisted of febrile individuals living in villages with malaria prevalence rates in 2–10 year olds ranging from 8% to 93% (S2 Fig). The malaria positivity rate in febrile individuals under the age of five increased from 0% in the lowest prevalence villages to 84% in the highest prevalence villages (Fig 2). A one standard deviation increase in the village prevalence rate was associated with 2.03 times higher odds that a febrile child under the age of five tested positive for malaria (95% CI: [1.63 2.54], p<0·001; Adjusted OR: 1.68, 95% CI: [1.31 2.16], p<0·001) (S2 Table). However, the likelihood that a febrile child was treated with an ACT does not increase with village prevalence (Fig 2). A one standard deviation increase in the village prevalence rate was associated with lower odds that a febrile episode in a child under 5 was treated with an ACT (Unadjusted OR: 0.83, 95% CI: [0.66 1.05], P = 0·113; Adjusted OR: 0.75, 95% CI: [0.60 0.93], P = 0·010) (S2 Table).

The odds that a clinical malaria episode (a fever with a positive RDT) was treated with an ACT was also not significantly associated with the age of the febrile individual age and was lower with increasing village malaria prevalence rates (S3 Fig, S1 Table and S2 Table).

### Malaria beliefs

**Fig 3:**
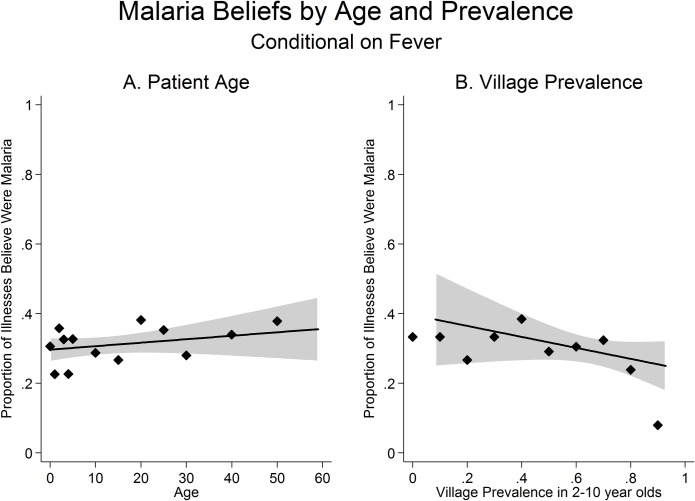
Malaria beliefs by age and prevalence. (A) The proportion of febrile illnesses reported as malaria by the age of the febrile individual. (B) The proportion of febrile illnesses reported as malaria by the village prevalence rate. Figure shows mean of the outcome within 1-year age groups (for children under 5) or 5-year age groups (for ages 5 and above) and 0.10 units of village prevalence. A local linear regression line is also plotted and the grey shaded areas indicate 95% confidence intervals. Sample is limited to individuals who had a fever in the two weeks prior to the survey and who were not previously tested for malaria. Ages above 60 are excluded because of small sample size. In Fig 3B, sample is limited to children under the age of 5.

We now turn from treatment of febrile illnesses to beliefs about whether the illness was malaria. Respondents reported 34% of febrile illnesses across all ages as malaria, with a slightly higher percent among febrile individuals aged 20–30 (Fig 3). Compared with febrile individuals ages 15 and above, respondents did not have higher odds of reporting that the illness was malaria for children ages 5–14 (Unadjusted OR: 1.04, 95%CI: [0.71 1.53], P = 0·829; Adjusted OR: 1.17, 95% CI: [0.80 1.70], P = 0·421) or children under the age of 5 (Unadjusted OR: 0·94, 95% CI: [0.72 1.24], P = 0·677; Adjusted OR: 0.97, 95% CI: [0.71 1.33], P = 0·863) (S3 Table). Similarly, Fig 3 suggests that beliefs about whether a febrile illness in a child under 5 was malaria were not associated with malaria prevalence. A one standard deviation increase in the village malaria prevalence rate was associated with 0.87 times the odds that a respondent identified a febrile illness in a child under 5 as malaria (95% CI: [0.70 1.08], P = 0·213, Adjusted OR: 0.89, 95% CI: [0.70 1.14], P = 0.365) (S3 Table).

### Factors associated with ACT treatment for febrile illnesses

Table 2 compares the magnitude of the bi-variate and multi-variate associations between respondent, febrile individual, and household characteristics and use of ACTs to treat a febrile illness (Columns 1 and 2) and ACT treatment of RDT-positive febrile episodes (Columns 3 and 4). A febrile individual who was believed by the respondent to have malaria had 2.54 times the odds of being treated with an ACT (95% CI: [1.86 3.47], P<0·001, Adjusted OR: 2.43, 95% CI: [1.78 3.33], P<0.001), which suggests that people’s perceptions of whether an illness is malaria are important in influencing the probability that a febrile individual is treated with an ACT. Similarly, a clinical malaria episode (an individual who had a fever and tested positive for malaria on the survey RDT) had 2.73 times higher odds of being treated with an ACT if the respondent believed the illness was malaria (95% CI: [1.83 4.07], P<0.001, Adjusted OR: 2.68, 95% CI: [1.79 4.01], P<0·001).

**Table 2:** Factors associated with ACT for febrile episodes and clinical malarial episodes.

	Febrile Episode Treated with ACT		RDT-Positive Febrile Episode Treated with ACT	
	OR	aOR	OR	aOR
Respondent Believed Illness Was Malaria	2.54**	2.43**	2.73**	2.68**
	[1.86,3.47]	[1.78,3.33]	[1.83,4.07]	[1.79,4.01]
Individual is Aged 15 and above	Ref. Group	Ref. Group	Ref. Group	Ref. Group
Child Aged 5–14	1.24	1.32	1.23	1.49
	[0.89,1.71]	[0.94,1.86]	[0.79,1.91]	[0.93,2.40]
Child Under Age 5	1.21	1.18	1.39	1.43
	[0.87,1.68]	[0.84,1.64]	[0.88,2.19]	[0.91,2.26]
Standardized Village Prevalence Rate	0.87	0.85*	0.84	0.73*
	[0.72,1.06]	[0.73,0.99]	[0.62,1.14]	[0.55,0.97]
Respondent Has No Education	Ref. Group	Ref. Group	Ref. Group	Ref. Group
Respondent Has Some Primary Education	2.31**	2.15**	1.98*	1.80*
	[1.51,3.55]	[1.38,3.36]	[1.15,3.41]	[1.06,3.07]
Respondent Has Some Secondary Education	3.02**	1.92	2.12*	1.44
	[1.67,5.47]	[0.93,3.96]	[1.02,4.40]	[0.65,3.20]
Respondent Can Read English	1.64**	1.18	1.37	1.29
	[1.21,2.21]	[0.77,1.81]	[0.93,2.01]	[0.79,2.10]
Household In First Wealth Quintile (Poorest)	Ref. Group	Ref. Group	Ref. Group	Ref. Group
Household In Second Wealth Quintile	1.22	1.06	1.35	1.08
	[0.79,1.88]	[0.67,1.65]	[0.79,2.31]	[0.64,1.85]
Household In Third Wealth Quintile	0.98	0.92	1.1	1.01
	[0.66,1.48]	[0.61,1.38]	[0.69,1.75]	[0.62,1.65]
Household In Fourth Wealth Quintile	1.61	1.5	1.09	1.13
	[0.95,2.71]	[0.90,2.51]	[0.55,2.14]	[0.60,2.11]
Household In Fifth Wealth Quintile (Richest)	1.84*	1.33	1.26	0.79
	[1.05,3.24]	[0.73,2.42]	[0.56,2.83]	[0.33,1.89]
Household distance to closest health center (km)	1	1.04	1	1.03
	[0.90,1.12]	[0.96,1.12]	[0.87,1.15]	[0.93,1.15]
Household distance to closest hospital (km)	0.99	1	0.98	0.98
	[0.96,1.01]	[0.98,1.02]	[0.94,1.02]	[0.95,1.01]
Household distance to closest clinic (km)	0.97	0.98	0.99	0.98
	[0.92,1.02]	[0.93,1.03]	[0.92,1.06]	[0.92,1.04]
Household distance to closest drug shop(km)	1.16*	1.16*	1.25**	1.23**
	[1.04,1.31]	[1.03,1.30]	[1.08,1.44]	[1.08,1.40]
ACTs available at closest licensed drug shop	1.36	1.47*	1.56	1.76*
	[0.89,2.08]	[1.07,2.00]	[0.83,2.92]	[1.08,2.86]
Proportion Treated with an ACT	0.4	0.4	0.39	0.39
Number of Obs	1103	1103	597	597

Notes: Columns 1 and 2 show associations with the odds of ACT treatment for a febrile illness, while Columns 3 and 4 present associations with the odds of ACT treatment for an RDT-positive febrile illness. Columns 1 and 3 are bi-variate regressions and columns 2 and 4 are multi-variate regressions. Confidence intervals are in brackets and adjusted for clustering at the village level.

*p<0.05

**p<0.01

In the multi-variate regression (Column 2), odds of ACT treatment for a febrile episode was higher if the respondent had some primary education (Adjusted OR:2.15, 95% CI: [1.38 3.36], P = 0.001), if ACTs were available at the closest drug shop (Adjusted OR:1.47, 95% CI: [1.07 2.00], P = 0.016) and as distance to the closest drug shop increased (Adjusted OR:1.16, 95% CI: [1.03 1.30], P = 0.012) with similar results for the odds of ACT treatment for RDT-positive febrile episodes (Column 4). In both the bi-variate and multi-variate regressions, age was not significantly associated with the odds of ACT treatment for both febrile episodes and clinical malaria episodes and in the multi-variate models, increasing village malaria prevalence was associated with statistically significant lower odds of ACT treatment. Results from using a stepwise approach to estimate the model are very similar (S4 Table).

## Discussion

Nearly 80% of children with malaria worldwide do not receive ACTs [1]. Using a household survey from Uganda, and the WHO methodology for appropriate treatment of malaria, we found that, although the age of the febrile individual and the village prevalence rate were strongly associated with malaria infection among febrile individuals, ACT treatment rates were not correspondingly higher among young children or in high-prevalence villages. Moreover, people’s beliefs about whether a febrile illness was malaria did not vary with the age of the febrile individual or with village prevalence, which suggests that household misperceptions about malaria risk may play an important role in the under-treatment of malaria.

We show that respondents’ beliefs about malaria are strongly associated with the odds that a febrile individual was treated with an ACT, even when controlling for other factors that might influence ACT treatment and be associated with beliefs about malaria likelihood. We also find associations between ACT treatment and whether the respondent had a primary education, whether ACTs were available at the closest drugs shop, and with distance to the closest drug shop. These results are consistent with previous studies indicating the importance of education and access to drugs in malaria treatment [26,31,34]. The positive association between distance to a drug shop and ACT treatment is, however, unexpected. While we do not have any evidence on why this is the case, one possible explanation is that households further from a drug shop may be more likely to visit a health facility and, therefore, have a higher likelihood of receiving ACT treatment.

It is possible that the inverse relationship we observe between the proportion of children treated with an ACT and village prevalence results from variations in ACT coverage across different villages. For example, villages with the highest ACT coverage rates could be expected to have the lowest prevalence of malaria due to potential effects of ACT treatment on reducing malaria transmission [35–38]. However, we did not find substantial variations across villages in the availability of ACTs at either public health facilities or at licensed drug shops–in villages across all prevalence levels, more than 90% of public health facilities had ACTs available at the time of the survey, while approximately 80% of licensed drug shops had ACTs available (S4 Fig). This suggests that the patterns of ACT treatment by village prevalence are not simply a result of variations in ACT coverage.

We also found that among those who received diagnostic testing for malaria, and tested positive, only 54% of sick individuals took an ACT (a much higher proportion- 91% took any type of anti-malarial drug). This indicates that additional efforts are needed to increase the availability and affordability of ACTs. Our results on malaria beliefs also suggest, however, that households may not seek malaria testing and treatment for fevers that they do not suspect to be malaria. This underscores the urgency of public health messaging that emphasizes the importance of all fevers being tested for malaria and receiving appropriate treatment for the illness. Furthermore, the messaging could focus on the importance of febrile individuals getting tested even when the caregiver believes the illness is unlikely to be malaria, because of the difficulty of identifying the disease on the basis of symptoms alone. In order for this policy to be successful, there will also need to be improved access to diagnostic testing, as well as interventions to raise confidence in malaria diagnostic testing which has varied across different contexts [7–9,39,40].

It is particularly concerning that children are under-treated with ACTs. Not only are children more likely than adults to have malaria, but the disease is more likely to develop into a severe and life-threatening illness in children [41]. Several studies have found no differences in treatment-seeking patterns, or in the probability of being treated with an ACT, by the age of the sick individual [27,30,31,42]. Our results suggest that this may be related to caregivers’ mis-perceptions about the relative likelihood of malaria in febrile children compared to febrile adults.

This study has some limitations. First, people who have developed immunity to the disease can harbor malaria parasites asymptomatically [43] and may test positive on the RDT even if their fever is caused by another illness. Since we do not have data on parasite densities or co-infection, we cannot identify the proportion of fevers that are attributable to malaria using logistic regression methods [44,45]. However, according to WHO guidelines, all individuals with a fever who test positive for malaria should be treated with ACTs and therefore this is the definition that we use to identify illnesses that should have received ACT treatment [2]. Furthermore, immunity to malaria disease generally develops with age, particularly in areas of high malaria transmission [25,43]. When comparing across villages with different malaria prevalences, we limit our analysis to children under the age of five for whom there is a consistent relationship between increasing prevalence and higher incidence of clinical malaria episodes [15].

Second, the malaria RDT we used in this study only detects infections caused by Plasmodium falciparum and, therefore, we may have missed infections that were caused by non-falciparum species [46]. However in Uganda, *P*. *falciparum* is responsible for between 90–97% of malaria cases [47].

Third, we relied on respondents’ self-reports of whether an individual had a fever in the two weeks prior to the survey and of whether the individual took an ACT. This may be affected by recall bias, social desirability bias and respondent knowledge of ACTs. By relying on respondents’ self-reports of whether the individual had a fever, we may have either under-estimated or over-estimate cases of clinical malaria.

Fourth, the respondent was asked about their beliefs about whether an illness was malaria in retrospect rather than prior to beginning treatment. It is possible that the treatment that was chosen for the illness, and the outcome of the illness, influenced their perceptions of whether the illness was malaria.

Finally, the generalizability of these results is limited by the fact that the survey occurred over a few months (March-May) in one part of Eastern Uganda. As households were surveyed over 9 weeks (the rainy season) it is unlikely that differences in measured prevalence rates are due to variations in season. However, the patterns of beliefs and behavior that we found in this study may vary by season or by location, and by the availability of ACTs relative to other anti-malarial drugs. Moreover, since this entire region is highly endemic for malaria, our results may not be relevant to low transmission and pre-elimination settings.

The likelihood that an individual with malaria is treated with an ACT depends on whether they (or their caregivers) decide to seek care, the availability and cost of ACTs locally, and their beliefs about the efficacy of those drugs [4,26,28,48]. Our results suggest that additional policies are needed to encourage caregivers to seek immediate diagnostic testing and treatment for febrile illnesses to ensure that those at highest risk of malaria receive appropriate care.

## Supporting information

S1 Dataset Household and illness survey. Household data from baseline survey and illness data from final survey round including malaria rapid diagnostic test result. Sample is limited to those who had a fever in the two weeks prior to the survey.
(DTA)

S1 Fig Robustness of malaria beliefs by age and prevalence. The figures in the left column show the proportion of respondents who reported that the illness was malaria. The figures on the right show the mean of the respondents’ perceived likelihood that the illness was malaria on a scale of 0–10 (divided by 10 so as to use a similar scale). Points show mean of the outcome within 5-year age groups or 0.1 units of village prevalence. A local linear regression line is also plotted and the grey shaded areas indicate 95% confidence intervals. Sample is limited to patients who were not previously tested for malaria.
(TIF)

S2 Fig Distribution of village malaria prevalence rates. Positivity Rates are based on the RDT performed at the end of the survey on children whose parents/caregivers gave consent to their being tested. Sample is limited to children between the ages of 2 and 10.
(TIF)

S3 Fig ACT use for clinical malaria episodes by age and by village prevalence. (A) The proportion of clinical malaria episodes (febrile patients who tested positive on the RDT) treated with ACTs by the age of the patient. (B) The proportion of clinical malaria episodes treated with ACTs by the village prevalence rate. Figure shows mean of the outcome within 1-year age groups (for ages under 5) or 5-year age groups (for ages 5 and above) and 0.10 units of village prevalence. A local linear regression line is also plotted and the grey shaded areas indicate 95% confidence intervals. Data is limited to patients who had a fever in the two weeks prior to the survey and to patients under age 5 (in B). Ages above 60 are excluded because of small sample size (in A).
(TIF)

S4 Fig ACT availability in health facilities and drugs shops by village prevalence. The proportion of health facilities (blue) and drug shops (red) by village prevalence that had ACTs available at the time of the survey. Health facilities include public clinics, health centers or hospitals. Points indicate mean proportion for each village and a separate local polynomial regression line is also included for health facilities and drug shops.
(TIF)

S1 Table Effect of age on malaria positivity and ACT use. Table shows un-adjusted and adjusted logistic regression results of the association between age and the odds that a febrile individual tested positive for malaria, the odds that a febrile individual was treated with an ACT, and the odds that an RDT-positive febrile episode was treated with an ACT. The control variables for the adjusted regressions are as follows: respondent’s education level, whether the respondent can read English, household wealth quintile, distance to closest clinic, health center, hospital and drug shop, and whether the closest licensed drug shop stocked ACTs. Equality of coefficients were tested using the “suest” command in STATA. 95% confidence intervals are in brackets and adjusted for clustering at the village level. *p<0.05, **p<0.01.
(DOCX)

S2 Table Effect of village prevalence on malaria positivity and ACT use among children under age 5. Table shows un-adjusted and adjusted logistic regression results of the association between the standardized village prevalence rate and the odds that a child under 5 tested positive for malaria, the odds that a child under 5 was treated with an ACT, and the odds that a clinical malaria episode (fever and RDT positive) in a child under 5 was treated with an ACT. Individual is excluded from calculation of village prevalence rate. The control variables for the adjusted regressions are as follows: respondent’s education level, whether the respondent can read English, household wealth quintile, distance to closest clinic, health center, hospital and drug shop, and whether the closest licensed drug shop stocked ACTs. Equality of coefficients were tested using the “suest” command in STATA. 95% confidence intervals are in brackets and adjusted for clustering at the village level. *p<0.05, **p<0.01.
(DOCX)

S3 Table Malaria beliefs by patient age and by village prevalence. Un-adjusted and adjusted logistic regression results of the association between patient age, or the village prevalence rate, and the odds that a respondent believed the febrile illness was malaria. Adjusted logistic regressions include the following control variables: respondent’s education level, whether the respondent can read English, household wealth quintile, distance to closest clinic, health center, hospital and drug shop, and whether the closest licensed drug shop stocked ACTs. Sample is limited to individuals who were not previously tested for malaria. Regressions with village prevalence are limited to children under the age of 5. Individuals who were previously tested for malaria are excluded. 95% confidence intervals are in brackets and are adjusted for clustering at the village level. *p<0.05, **p<0.01.
(DOCX)

S4 Table Factors associated with ACT treatment using stepwise multivariate logistic regression. Results are from a stepwise backward-selection estimation of a logistic model (using the “stepwise” command in STATA). The significance level for removal from the model was 0.2. 95% confidence intervals are in brackets and are adjusted for clustering at the village level. *p<0.05, **p<0.01.
(DOCX)
