# Supplementary figures and images for: Disparities between malaria infection and treatment rates: Evidence from a cross-sectional analysis of households in Uganda
Source: PLoS One. 2017 Feb 27;12(2):e0171835. doi: 10.1371/journal.pone.0171835 (PMC5328248; doi:10.1371/journal.pone.0171835)

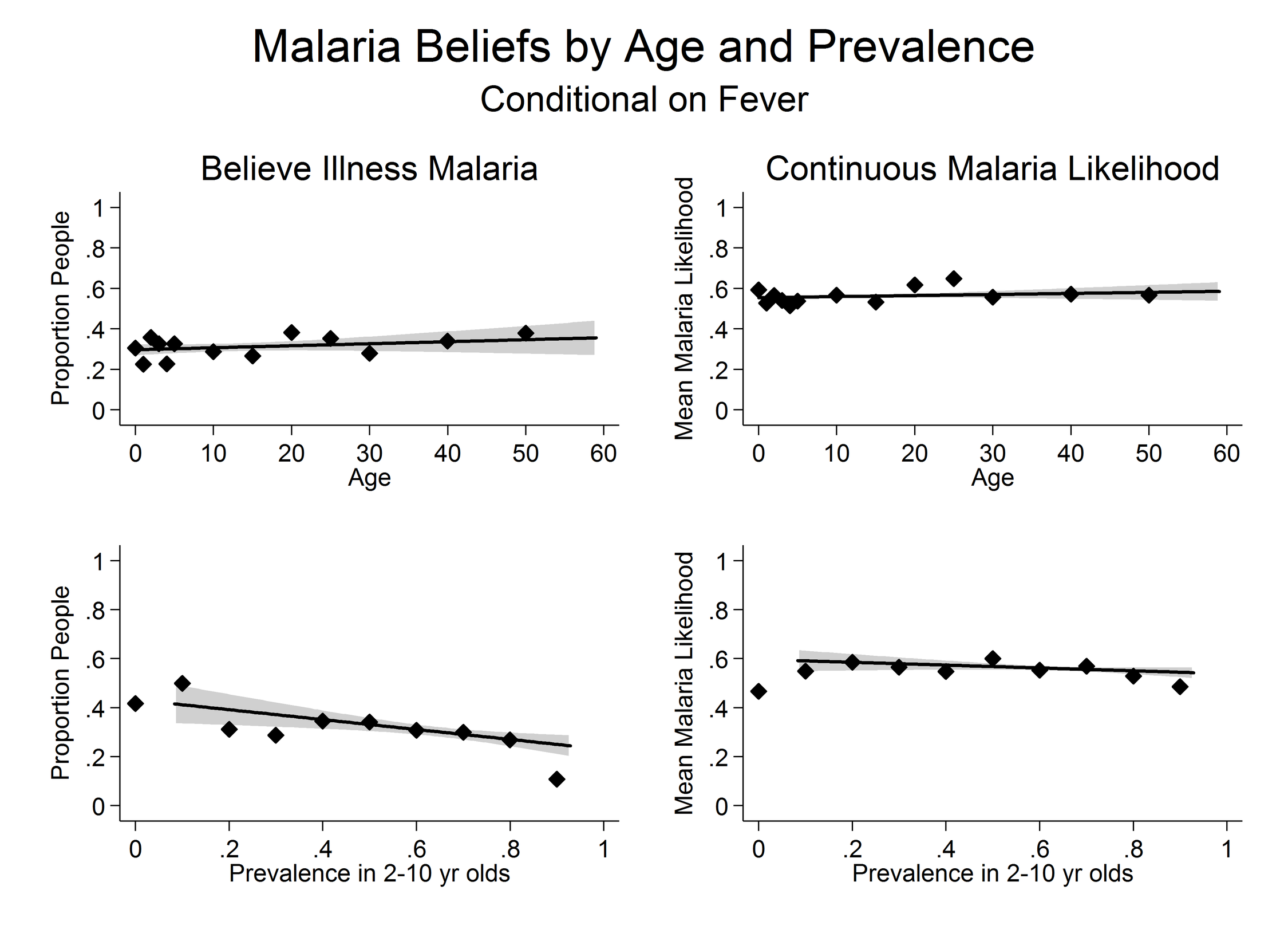

Supplement: S1 Fig — The figures in the left column show the proportion of respondents who reported that the illness was malaria. The figures on the right show the mean of the respondents’ perceived likelihood that the illness was malaria on a scale of 0–10 (divided by 10 so as to use a similar scale). Points show mean of the outcome within 5-year age groups or 0.1 units of village prevalence. A local linear regression line is also plotted and the grey shaded areas indicate 95% confidence intervals. Sample is limited to patients who were not previously tested for malaria. (TIF) [file pone.0171835.s002.tif]

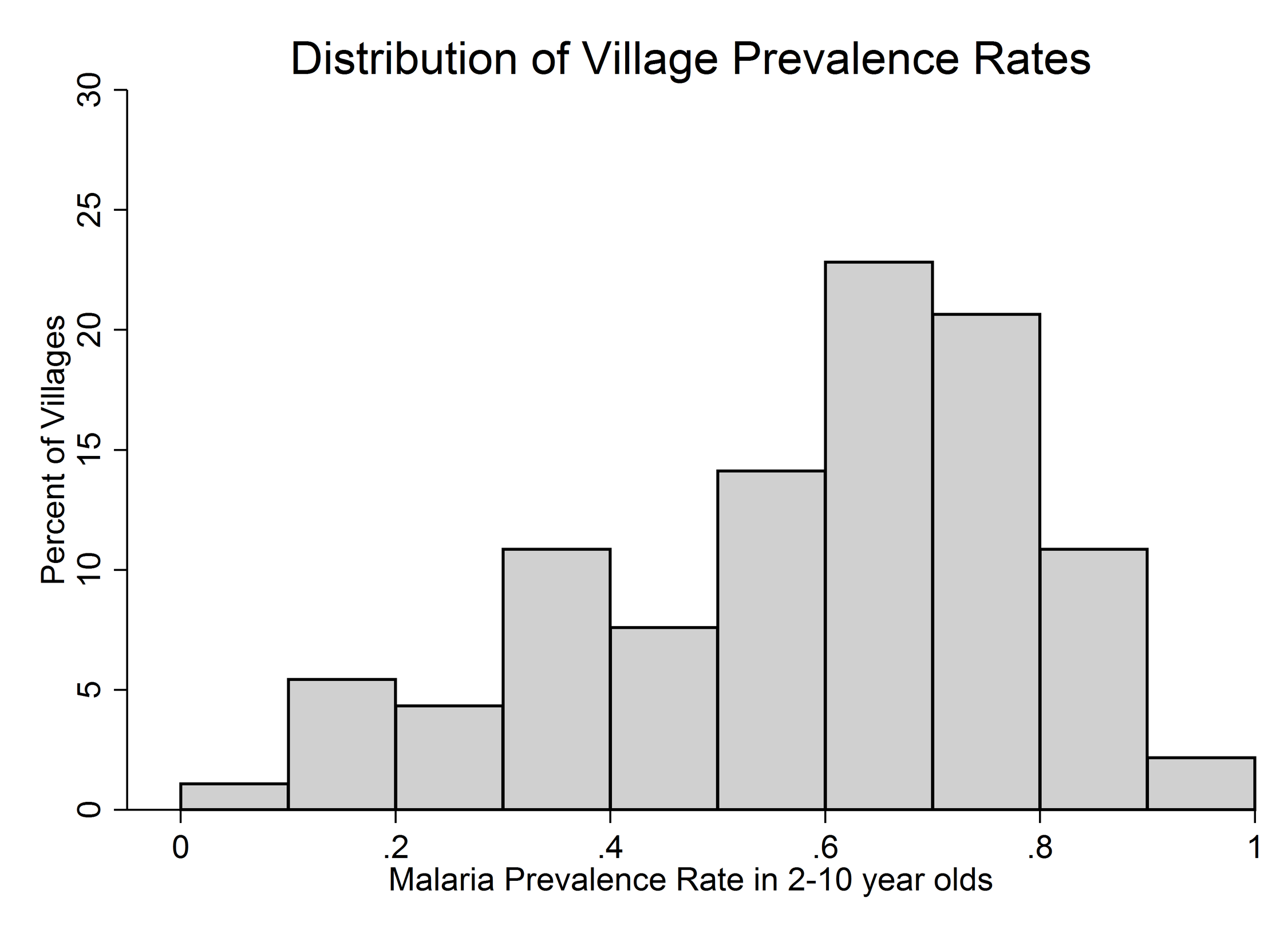

Supplement: S2 Fig — Positivity Rates are based on the RDT performed at the end of the survey on children whose parents/caregivers gave consent to their being tested. Sample is limited to children between the ages of 2 and 10. (TIF) [file pone.0171835.s003.tif]

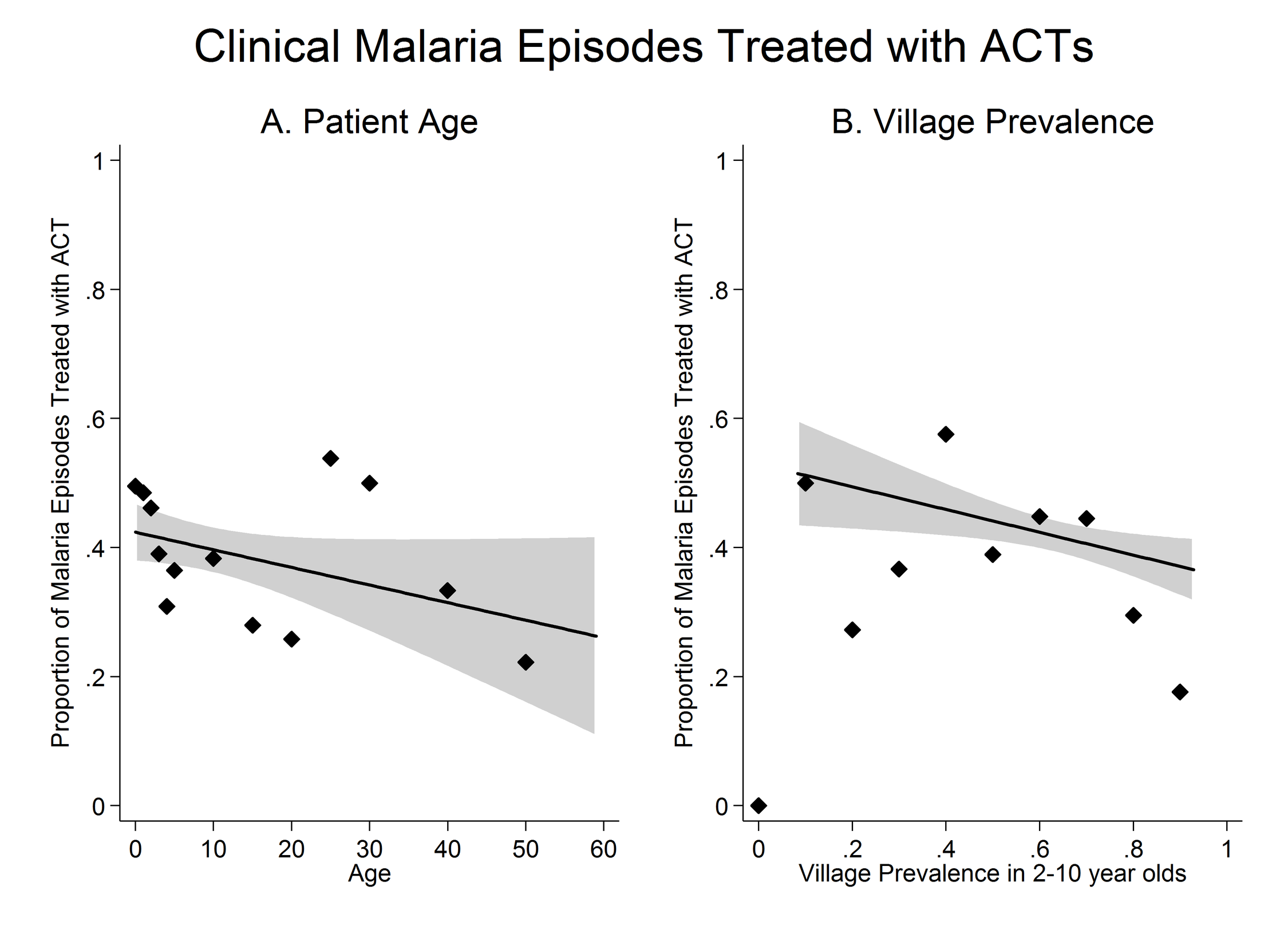

Supplement: S3 Fig — (A) The proportion of clinical malaria episodes (febrile patients who tested positive on the RDT) treated with ACTs by the age of the patient. (B) The proportion of clinical malaria episodes treated with ACTs by the village prevalence rate. Figure shows mean of the outcome within 1-year age groups (for ages under 5) or 5-year age groups (for ages 5 and above) and 0.10 units of village prevalence. A local linear regression line is also plotted and the grey shaded areas indicate 95% confidence intervals. Data is limited to patients who had a fever in the two weeks prior to the survey and to patients under age 5 (in B). Ages above 60 are excluded because of small sample size (in A). (TIF) [file pone.0171835.s004.tif]

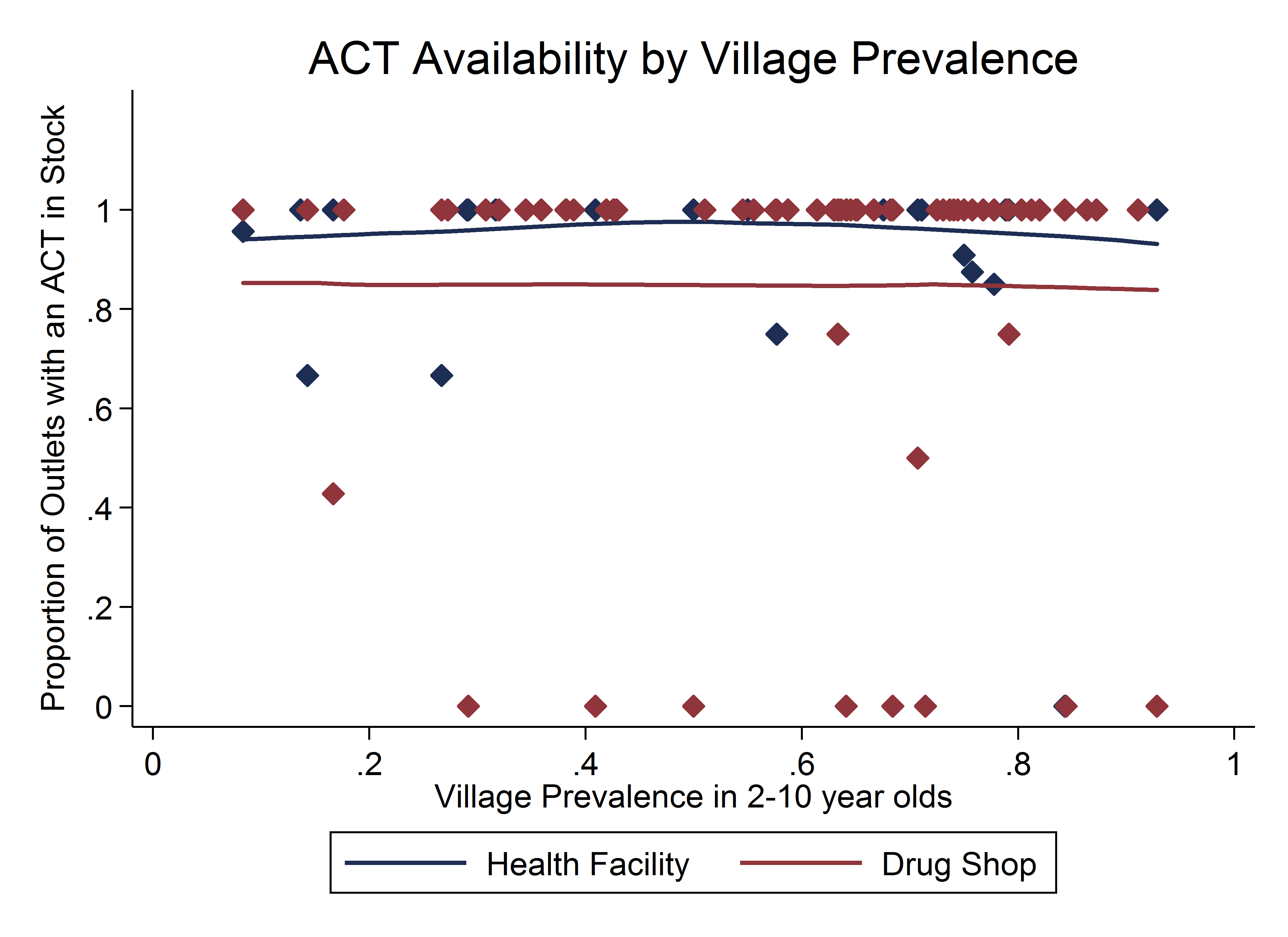

Supplement: S4 Fig — The proportion of health facilities (blue) and drug shops (red) by village prevalence that had ACTs available at the time of the survey. Health facilities include public clinics, health centers or hospitals. Points indicate mean proportion for each village and a separate local polynomial regression line is also included for health facilities and drug shops. (TIF) [file pone.0171835.s005.tif]
