# Supplementary material for: Disparities between malaria infection and treatment rates: Evidence from a cross-sectional analysis of households in Uganda
Source: PLoS One. 2017 Feb 27;12(2):e0171835. doi: 10.1371/journal.pone.0171835 (PMC5328248; doi:10.1371/journal.pone.0171835)
Supplement: S1 Table — Table shows un-adjusted and adjusted logistic regression results of the association between age and the odds that a febrile individual tested positive for malaria, the odds that a febrile individual was treated with an ACT, and the odds that an RDT-positive febrile episode was treated with an ACT. The control variables for the adjusted regressions are as follows: respondent’s education level, whether the respondent can read English, household wealth quintile, distance to closest clinic, health center, hospital and drug shop, and whether the closest licensed drug shop stocked ACTs. Equality of coefficients were tested using the “suest” command in STATA. 95% confidence intervals are in brackets and adjusted for clustering at the village level. *p<0.05, **p<0.01. (DOCX) [file pone.0171835.s006.docx]

**S1 Table. Effect of Age on Malaria Positivity and ACT Use.**

Table shows un-adjusted and adjusted logistic regression results of the association between age and the odds that a febrile individual tested positive for malaria, the odds that a febrile individual was treated with an ACT, and the odds that an RDT-positive febrile episode was treated with an ACT. The control variables for the adjusted regressions are as follows: respondent’s education level, whether the respondent can read English, household wealth quintile, distance to closest clinic, health center, hospital and drug shop, and whether the closest licensed drug shop stocked ACTs. Equality of coefficients were tested using the “suest” command in STATA. 95% confidence intervals are in parentheses and are adjusted for clustering at the village level. *p<0.05, **p<0.01.
