# Supplementary material for: Disparities between malaria infection and treatment rates: Evidence from a cross-sectional analysis of households in Uganda
Source: PLoS One. 2017 Feb 27;12(2):e0171835. doi: 10.1371/journal.pone.0171835 (PMC5328248; doi:10.1371/journal.pone.0171835)
Supplement: S2 Table — Table shows un-adjusted and adjusted logistic regression results of the association between the standardized village prevalence rate and the odds that a child under 5 tested positive for malaria, the odds that a child under 5 was treated with an ACT, and the odds that a clinical malaria episode (fever and RDT positive) in a child under 5 was treated with an ACT. Individual is excluded from calculation of village prevalence rate. The control variables for the adjusted regressions are as follows: respondent’s education level, whether the respondent can read English, household wealth quintile, distance to closest clinic, health center, hospital and drug shop, and whether the closest licensed drug shop stocked ACTs. Equality of coefficients were tested using the “suest” command in STATA. 95% confidence intervals are in brackets and adjusted for clustering at the village level. *p<0.05, **p<0.01. (DOCX) [file pone.0171835.s007.docx]

**S2 Table. Effect of Village Prevalence on Malaria Positivity and ACT Use among Children Under Age 5.**

Table shows un-adjusted and adjusted logistic regression results of the association between the standardized village prevalence rate and the odds that a febrile child under 5 tested positive for malaria, the odds that a febrile cdhild under 5 was treated with an ACT, and the odds that a clinical malaria episode (fever and RDT positive) in a child under 5 was treated with an ACT. Individual is excluded from calculation of village prevalence rate. The control variables for the adjusted regressions are as follows: respondent’s education level, whether the respondent can read English, household wealth quintile, distance to closest clinic, health center, hospital and drug shop, and whether the closest licensed drug shop stocked ACTs. Equality of coefficients were tested using the “suest” command in STATA. 95% confidence intervals are in parentheses and are adjusted for clustering at the village level. *p<0.05, **p<0.01.
