# Supplementary material for: Disparities between malaria infection and treatment rates: Evidence from a cross-sectional analysis of households in Uganda
Source: PLoS One. 2017 Feb 27;12(2):e0171835. doi: 10.1371/journal.pone.0171835 (PMC5328248; doi:10.1371/journal.pone.0171835)
Supplement: S3 Table — Un-adjusted and adjusted logistic regression results of the association between patient age, or the village prevalence rate, and the odds that a respondent believed the febrile illness was malaria. Adjusted logistic regressions include the following control variables: respondent’s education level, whether the respondent can read English, household wealth quintile, distance to closest clinic, health center, hospital and drug shop, and whether the closest licensed drug shop stocked ACTs. Sample is limited to individuals who were not previously tested for malaria. Regressions with village prevalence are limited to children under the age of 5. Individuals who were previously tested for malaria are excluded. 95% confidence intervals are in brackets and are adjusted for clustering at the village level. *p<0.05, **p<0.01. (DOCX) [file pone.0171835.s008.docx]

**S3 Table. Malaria Beliefs by Patient Age and by Village Prevalence**

Un-adjusted and adjusted logistic regression results of the association between patient age, or the village prevalence rate, and the odds that a respondent believed the febrile illness was malaria. Adjusted logistic regressions include the following control variables: respondent’s education level, whether the respondent can read English, household wealth quintile, distance to closest clinic, health center, hospital and drug shop, and whether the closest licensed drug shop stocked ACTs. Sample is limited to individuals who were not previously tested for malaria. Regressions with village prevalence are limited to children under the age of 5. Individuals who were previously tested for malaria are excluded. 95% confidence intervals are in brackets and are adjusted for clustering at the village level. *p<0.05, **p<0.01.
