# Supplementary material for: Disparities between malaria infection and treatment rates: Evidence from a cross-sectional analysis of households in Uganda
Source: PLoS One. 2017 Feb 27;12(2):e0171835. doi: 10.1371/journal.pone.0171835 (PMC5328248; doi:10.1371/journal.pone.0171835)
Supplement: S4 Table — Results are from a stepwise backward-selection estimation of a logistic model (using the “stepwise” command in STATA). The significance level for removal from the model was 0.2. 95% confidence intervals are in brackets and are adjusted for clustering at the village level. *p<0.05, **p<0.01. (DOCX) [file pone.0171835.s009.docx]

**S4 Table : Factors Associated with ACT treatment using Stepwise Multivariate Logistic Regression**

|  | Stepwise Logistic Regression | |
| --- | --- | --- |
|  | Odds Febrile Episode Treated with ACT | Odds RDT-Positive Febrile Episode Treated with ACT |
|  | (1) | (2) |
| Respondent Believed Illness Was Malaria | 2.47** | 2.65** |
|  | [1.82,3.34] | [1.80,3.92] |
|  |  |  |
| Patient is Aged 15 and above | Ref. Group | Ref. Group |
|  |  |  |
|  |  |  |
| Patient Aged 5-14 |  | 1.45 |
|  |  | [0.90,2.34] |
|  |  |  |
| Patient Under Age 5 |  | 1.42 |
|  |  | [0.89,2.28] |
|  |  |  |
| Standardized Village Prevalence Rate | 0.86 | 0.74* |
|  | [0.73,1.01] | [0.55,1.00] |
|  |  |  |
| Respondent Has No Education | Ref. Group | Ref. Group |
|  |  |  |
|  |  |  |
| Respondent Has Some Primary Education | 2.24** | 1.90* |
|  | [1.45,3.44] | [1.11,3.24] |
|  |  |  |
| Respondent Has Some Secondary Education | 2.24** | 1.78 |
|  | [1.24,4.05] | [0.88,3.60] |
|  |  |  |
| Respondent Can Read English |  |  |
|  |  |  |
|  |  |  |
| Household In First Wealth Quintile (Poorest) | Ref. Group | Ref. Group |
|  |  |  |
|  |  |  |
| Household In Second Wealth Quintile |  |  |
|  |  |  |
|  |  |  |
| Household In Third Wealth Quintile |  |  |
|  |  |  |
|  |  |  |
| Household In Fourth Wealth Quintile | 1.54* |  |
|  | [1.01,2.33] |  |
|  |  |  |
| Household In Fifth Wealth Quintile (Richest) | 1.39 |  |
|  | [0.88,2.20] |  |
|  |  |  |
| Household distance to closest health center (km) |  |  |
|  |  |  |
|  |  |  |
| Household distance to closest hospital (km) |  | 0.98 |
|  |  | [0.95,1.01] |
|  |  |  |
| Household distance to closest clinic (km) |  |  |
|  |  |  |
|  |  |  |
| Household distance to closest drug shop(km) | 1.15* | 1.20** |
|  | [1.02,1.28] | [1.06,1.37] |
|  |  |  |
| ACTs available at closest licensed drug shop | 1.50** | 1.74* |
|  | [1.11,2.04] | [1.10,2.76] |
|  |  |  |
| Proportion of people who took an ACT | 0.4 | 0.39 |
| Number of Observations | 1103 | 597 |

Notes: Results are from a stepwise backward-selection estimation of a logistic model (using the “stepwise” command in STATA). The significance level for removal from the model was 0.2. 95% confidence intervals are in brackets and are adjusted for clustering at the village level. *p<0.05, **p<0.01
